# Rat models of musculoskeletal lysosomal storage disorders and their role in pre-clinical evaluation of gene therapy approaches

**DOI:** 10.1007/s00335-025-10121-3

**Published:** 2025-03-18

**Authors:** Sara Marcó, Sergio Muñoz, Fatima Bosch, Veronica Jimenez

**Affiliations:** 1 Center of Animal Biotechnology and Gene Therapy, Department of Biochemistry and Molecular Biology, School of Veterinary Medicine, Universitat Autònoma de Barcelona, Bellaterra, Spain; 2 Centro de Investigación Biomédica en Red de Diabetes y Enfermedades Metabólicas Asociadas (CIBERDEM), Madrid, Spain

**Keywords:** Rat model, Lysosomal storage disorders, Pompe disease, Danon disease, Morquio A syndrome, Maroteaux-Lamy syndrome, AAV vectors

## Abstract

Mice have been a cornerstone of biomedical research for decades for studying a wide range of biological processes, disease mechanisms, and the assessment of therapies. Moreover, mice present several practical advantages such as small size, low cost and ease of genetic manipulation. While mice offer numerous benefits, for certain disease areas, rat models provide a closer representation of human disease progression, offering better insights for translational research and therapeutic development. This closer resemblance is particularly important for research focusing on diseases involving the cardiovascular and musculoskeletal system. In rats, the pathophysiology of these diseases mirrors the clinical alterations observed in humans. This review focuses on the key phenotypic differences between mouse and rat models of lysosomal storage disorders that specifically manifest with cardiac, skeletal muscle, and bone and joint involvement (Pompe and Danon diseases, and Maroteaux-Lamy and Morquio A syndromes). Furthermore, we discuss the therapeutic potential of various adeno-associated viral vector-mediated gene therapies that have been evaluated in these rat models, highlighting their contributions to advancing treatment options for these debilitating conditions.

## Introduction

The development of animal models that replicate human diseases is essential for understanding their molecular basis and testing new therapies. Rats are the second most used mammal in biomedical research, after mice (Hickman et al. 2017). Domesticated in the 1700s, the laboratory rat, *Rattus norvegicus*, has been used in research since 1828, with major efforts starting at the Wistar Institute in 1894 (Hedrich 2000; Fox 2002; Lindsey and Baker 2006). Though once dominant in research, mice became more common in recent decades due to their small size, low operational costs, ease of isolation and culture of mouse embryonic stem (ES) cells together with their relative stability, and the pliability for various genetic manipulations and gene editing in mice (Iannaccone and Jacob 2009; Zheng et al. 2012). These practical advantages have facilitated extensive research across various biological disciplines. However, while mice are extremely valuable tools, their physiology and disease manifestations can differ significantly from certain human conditions.

Currently, the rat genome and chromosome mapping technologies have been developed, the genomic sequence of dozens of rat strains has been established, and several resources providing genetic, genomic, phenotype and disease-relevant data in rats are available (e.g. Rat Genome Database (RGD); PhenoMiner; PhysGen Knockout program; Rat Genome Resources at National Center for Biotechnology Information; MCW Gene Editing Rat Resource Centre; Rat Resource and Research Centre (RRRC); National Bio Resource Project for the Rat (NBRP-Rat); European large-scale functional genomics in the rat for translational research (EURATRANS); Research Resource Identifier portal (RRID) (SERIKAWA et al. 2009; Laulederkind et al. 2013, 2023; Wang et al. 2015; Shimoyama et al. 2017; Meek et al. 2017; Zinski et al. 2021). Moreover, rat ES cells can be efficiently derived, and genetic modification technologies (including gene editing by means of TALENs, ZFN and CRISPR/Cas9) have been successfully developed in rats (Ying et al. 2008; Buehr et al. 2008; Li et al. 2008, 2013; Geurts et al. 2009; Kawamata and Ochiya 2010; Tong et al. 2010, 2011; Tesson et al. 2011; Bertolin et al. 2021; Muñoz et al. 2024), increasing the use of rats in biomedicine, particularly for certain research areas in which rats better reproduce human diseases.

Rats are particularly valuable in neuroscience, where they exhibit more complex cognitive abilities and social behavior than mice (Iannaccone and Jacob 2009; Parker et al. 2014; Ellenbroek and Youn 2016). Rat models of breast cancer, diabetes, cardiovascular disease, and other disorders closely resemble human conditions, showing better hormone responsiveness and disease progression (Iannaccone and Jacob 2009; Sanders and Samuelson 2014; Ma et al. 2018; Perez-Leighton et al. 2020; Nicotra et al. 2024). They are widely used in pharmacology and toxicology due to their similar detoxification pathways to humans (Hashway and Wilding 2020). Noticeably, rat models of a wide variety of disorders, including diseases with musculoskeletal involvement, show disease phenotypes that more closely recapitulate the pathological clinical features of the human disease, even when compared with mouse models with equivalent genetic modifications (Hammer et al. 1990; von Horsten et al. 2003; Alam et al. 2004; Yamada et al. 2004; Lo Bianco et al. 2004; Tessitore et al. 2008; Liu et al. 2008; Larcher et al. 2014; Lambert et al. 2016; Szpirer 2020; Bertolin et al. 2021; Nakamura et al. 2023; Muñoz et al. 2024).

The rat genome is more similar to humans than that of mice, not only in terms of genome length and number of chromosomes but in the percentage of genes that humans share with rats (99%) (Tesson et al. 2005). Moreover, the rat genome demonstrates a higher conservation of disease-related genes (Tesson et al. 2005; Szpirer 2020). These genomic differences may contribute to the greater physiological similarities observed between rats and humans in comparison with mice.

Additionally, rats’ physiology has extensively been studied, and rats’ larger size offers practical benefits for surgery and sampling, and for electrophysiology and imaging technologies (Iannaccone and Jacob 2009). These attributes make rats particularly useful in translational research, such as gene transfer and drug administration studies (Alam et al. 2004; Lo Bianco et al. 2004; Tessitore et al. 2008; Iannaccone and Jacob 2009; Jackson et al. 2015; Dayton et al. 2018; Bertolin et al. 2021; Muñoz et al. 2024; Baine et al. 2024).

## Lysosomal storage disorders

Lysosomal storage disorders (LSDs) encompass a group of more than 70 inborn errors of metabolism caused by mutations in genes encoding a lysosomal enzyme involved in normal lysosomal function (Parenti et al. 2015; Ferreira and Gahl 2023). Individually, each LSD is rare, or in some cases even ultra-rare, but the combined birth incidence as a group can range from 1 in 5,000 to 1 in 8,000 individuals (Ferreira and Gahl 2023). Deficiency of lysosomal enzymes results in accumulation of undegraded substrates that leads to the dysregulation of several cellular processes linked to this organelle, such as autophagy, exocytosis, membrane repair, lipid homeostasis, signaling cascades, or cell viability (Marques and Saftig 2019). Clinically, the affected patients show a variety of clinical signs and symptoms depending on the organs and tissues where undegraded substrates get accumulated (Ferreira and Gahl 2023). In general, LSD are multi-systemic disorders that lead to an early death (Pastores 2008). Although these diseases are primarily found in the pediatric population, most LSDs are characterized by a wide range in age of onset and diagnosis might not be apparent in attenuated variants which present in adulthood (Pastores 2008). Neurodegenerative features and musculoskeletal complications are often seen in the most severe variants and are features of the disease that have the most significant impact on patients’ physical and functional well-being (Pastores 2008).

This review examines LSDs that either exhibit significant skeletal and cardiac muscle pathology or are characterized by distinctive bone and joint abnormalities, with a specific emphasis on disorders for which mouse and rat research models have been established (Pompe and Danon diseases, and Maroteaux-Lamy and Morquio A syndromes).

### Pompe disease

Pompe disease (PD) is a rare metabolic autosomal recessive lysosomal storage disease caused by mutations in the gene coding for acid alpha-glucosidase (GAA) (van der Ploeg and Reuser 2008). The absence of this lysosomal enzyme leads to accumulation of undegraded glycogen, mainly in skeletal and cardiac muscle, causing progressive severe myopathy (van der Ploeg and Reuser 2008). Infantile-onset Pompe disease (IOPD) is caused by a severe or complete GAA deficiency with < 1% residual enzyme activity (Stevens et al. 2022). Clinical features are related to neonatal onset and rapidly progressive muscular pathology, leading to skeletal muscle weakness and hypotonia, cardiomegaly and respiratory failure, due to the malfunctioning of diaphragm and respiratory muscles (van der Ploeg and Reuser 2008). In IOPD, death generally occurs during the first year of life due to a cardiorespiratory failure, although the less severe forms have longer life expectancy (van der Ploeg and Reuser 2008). Late-onset Pompe disease (LOPD) is caused by a partial deficiency (< 30% residual activity) of GAA (Stevens et al. 2022). Patients with LOPD have a more slowly progressive skeletal myopathy eventually resulting in mobility problems and respiratory difficulties but generally do not present with hypertrophic cardiomyopathy (van der Ploeg and Reuser 2008).

To date, seven different PD mouse models have been developed (Raben et al. 1998, 2000; Bijvoet et al. 1998; Colella et al. 2020; Huang et al. 2020; Kan et al. 2022). Five of these PD models are knock-out (KO) mice in which different exons of the *Gaa* gene have been targeted. The other two models are knock-in (KI) mice harboring human *Gaa* mutations. The first KO mouse models were generated by targeted disruption of the *Gaa* gene by the insertion of the neomycin-resistance gene in exon 6 or 13 (6^neo^/6^neo^ mice [B6;129-*Gaa*^tm1Rabn^/J; MGI:3033756; RRID: IMSR_JAX:004154] and 13^neo^/13^neo^ mice [B6;FVB-*Gaa*^tm1Vdp^; MGI:3619140]) (Raben et al. 1998; Bijvoet et al. 1998). Subsequently, mutant mice in which exon 6 of the *Gaa* gene and the neomycin-resistance gene were removed by Cre/lox-mediated recombination (D6/D6 mice [129 × 1/SvJ; C57BL/6;FVB/N-*Gaa*^tm1.1Rabn^; MGI:3624424]) were generated (Raben et al. 1998). In another KO mouse model, exon 14 of the *Gaa* gene was replaced by the neomycin-resistance gene (D14^neo^/D14^neo^ mice [129 × 1/SvJ; C57BL/6-*Gaa*^tm2Rabn^; MGI:3624423]) (Raben et al. 2000). Recently, a novel Gaa KO mouse model of PD was generated by crossing the 6^neo^/6^neo^ mice with wild-type (WT) DBA2/J mice (Gaa KO^DBA2/J^ mice [B6.129;D2-Gaa^tm1Fmin^]) (Colella et al. 2020). The DBA2/J background, harboring the Latent TGF-β-binding protein 4 (Ltbp4)^D36^ allele, has been described to act as a modulator of the severity of Duchenne muscular dystrophy in mice and humans (Colella et al. 2020). The two KI mouse models of PD were generated using CRISPR-Cas9 technology. In these mice, the c.1826dupA (p.Y609*) or the c.1935 C > A (p.D645E) mutations, which cause human IOPD, were introduced in exon 13 or 14 of the *Gaa* gene, respectively (Gaa^c.1826dupA^ mice [C57BL/6NJ-*Gaa*^em1Jhng^/J; MGI:6454667; RRID: IMSR_JAX:034609] and Gaa^em1935C> A^ mice [C57BL/6NJ-*Gaa*^em2Jhng^/J; MGI:7327566; RRID: IMSR_JAX:037502]) (Huang et al. 2020; Kan et al. 2022).

All these mouse models accumulated glycogen in skeletal muscles and the heart but showed variable disease severity. The 6^neo^/6^neo^ mice exhibited early-onset myopathy and severely impaired muscle function (Raben et al. 1998). They are the most widely used PD animal model to study and characterize the disease pathophysiology, underlying molecular mechanisms, disease biomarkers, and for pre-clinical therapy development over the past 25 years. Nevertheless, 6^neo^/6^neo^ mice developed critical features of both the infantile and adult forms of the human Pompe disease (Raben et al. 1998; Puzzo et al. 2017; Keeler et al. 2019; Cagin et al. 2020; Colella et al. 2020; Clarke et al. 2021). Recently, a new KI rat model of PD was generated by introducing the specific inactivating R385STOP nonsense point mutation (C > T SNP) in exon 7 (catalytic site) of the *Gaa* gene by CRISPR/Cas9 technology (SD-*Gaa*^em1Fbos^) (Muñoz et al. 2024).

Both 6^neo^/6^neo^ mouse and PD rat models of Pompe disease lacked GAA enzyme activity and exhibited progressive glycogen accumulation in skeletal muscles starting very early in life (3–4 weeks of age) (Raben et al. 1998; Muñoz et al. 2024). However, the rat model demonstrated a more rapid and severe disease progression. While both models initially accumulated glycogen within lysosomes, PD rats showed earlier accumulation in the sarcoplasm as well, a feature linked to disease severity in humans (Raben et al. 1998; Thurberg et al. 2006; Schänzer et al. 2017; Muñoz et al. 2024). Moreover, the rat model exhibited earlier onset of other pathological hallmarks, including glycogen accumulation in the diaphragm, sarcoplasmic vacuolization and fragmentation, myofiber necrosis, impaired muscle function, reduced muscle weight, and growth retardation compared to 6^neo^/6^neo^ mice (Raben et al. 1998; Keeler et al. 2019; Colella et al. 2020; Muñoz et al. 2024). These findings indicated that PD rats develop a more aggressive form of the disease, more closely resembling the severe pathology observed in human IOPD (Slonim et al. 2000; van den Hout et al. 2003; Marsden 2005; Kishnani et al. 2006; Muñoz et al. 2024).

PD rats also showed early lethality. Whereas 6^neo^/6^neo^ mice showed a mean survival of 9–10 months and maximal survival of 18–24 months (Puzzo et al. 2017; Keeler et al. 2019; Cagin et al. 2020; Colella et al. 2020; Clarke et al. 2021), mean survival of PD rats was ~ 4.5 months, with no PD rats surviving over 8 months.

The 6^neo^/6^neo^ mouse model was similar to D6/D6 and 13^neo^/13^neo^ mice with respect to the level of enzyme activity and absence of GAA protein (Raben et al. 1998; Bijvoet et al. 1998). Although D6/D6 and 13^neo^/13^neo^ mice showed increased accumulation of glycogen, D6/D6 mice showed unimpaired muscular strength and mobility and 13^neo^/13^neo^ mice lacked overt clinical symptoms, except for cardiomegaly and abnormal electrocardiogram at 32 weeks of age (Raben et al. 1998; Bijvoet et al. 1998). Therefore, unlike human patients and PD rats, D6/D6 and 13^neo^/13^neo^ mice did not show clinical signs of muscle weakness.

In the D14^neo^/D14^neo^, Gaa KO^DBA2/J^, Gaa^c.1826dupA^ and Gaa^em1935C> A^ mouse models, glycogen accumulation was reported between 3 and 7 months of age but neither sarcoplasm vacuolization nor fragmentation were observed in skeletal muscle (Raben et al. 2000; Colella et al. 2020; Huang et al. 2020; Kan et al. 2022). In contrast, decreased muscle weight and weakness, and growth retardation were more pronounced in PD rats (Raben et al. 2000; Colella et al. 2020; Huang et al. 2020; Kan et al. 2022). Only Gaa KO^DBA2/J^ mice presented anticipated mortality, with ~ 50% survival at 6 months of age (Colella et al. 2020). Nevertheless, whether the exacerbated phenotype observed in Gaa KO^DBA2/J^ mice is an artifact because of increased TGFβ release due to the Ltbp4^D36^ allele in the DBA2/J background remains to be elucidated. Moreover, the role of LTBP4 in IOPD human etiology has not been proved.

Similar to IOPD patients, PD rats presented hypertrophic cardiomyopathy and hepatomegaly at young ages (Slonim et al. 2000; van den Hout et al. 2003; Marsden 2005; Kishnani et al. 2006). PD rats exhibited the earliest onset of cardiomegaly, with increased heart weight already detected at 2 months of age. In contrast, cardiomegaly was reported between 3 and 8 months of age in PD mouse models (Bijvoet et al. 1998; Colella et al. 2019, 2020; Huang et al. 2020; Kan et al. 2022).

In summary, the PD rats are the PD murine model that most closely mimics the human IOPD phenotype since they show the earliest onset of the main IOPD clinical symptoms and the lowest survival rate in comparison with any existing PD mouse model. The therapeutic efficacy against IOPD of an innovative muscle-directed adeno-associated viral (AAV) vector-mediated gene therapy has been recently assessed in these rats (Muñoz et al. 2024). Treatment of PD rats with an AAV vector designed to efficiently target expression of GAA in muscle resulted in highly efficient transduction of cardiac and skeletal muscles throughout the body, thus increasing the levels of GAA activity, normalizing glycogen storage pathology, restoring body weight, muscle mass and strength, counteracting cardiomegaly and normalizing survival rate (Muñoz et al. 2024). These results indicated that this gene therapy holds great potential to treat glycogen metabolism alterations in IOPD.

### Danon disease

Danon disease (DD) is a rare, X-linked dominant disorder characterized by cardiomyopathy, skeletal muscle weakness, and intellectual disability (Saftig et al. 2001; Zhai et al. 2023). It is caused by mutations in the LAMP2 gene, which disrupts autophagy and leads to accumulation of abnormal autophagic vacuoles and glycogen in skeletal and cardiac muscle cells (D’souza et al. 2014; Endo et al. 2015; Rowland et al. 2016; Cenacchi et al. 2020). Males typically experience more severe symptoms with earlier onset compared to females due to X-chromosome inactivation (D’souza et al. 2014). Prognosis is poor for male patients, often requiring heart transplantation, while females generally have a milder course with later onset of cardiomyopathy (Boucek et al. 2011; Endo et al. 2015; Sugie et al. 2018; Brambatti et al. 2019; Cenacchi et al. 2020; Zhai et al. 2023). Male patients usually die during adolescence, whereas females typically survive into their 30–40 s (Boucek et al. 2011).

To date, two DD mouse models have been developed (Tanaka et al. 2000; Alcalai et al. 2021). The first KO mouse model was generated by gene-targeting disruption of the LAMP2 gene (129P2/OlaHsd-*Lamp2*^tm1Psa^; MGI:6259615) (Tanaka et al. 2000). This animal model recapitulated some features of the human disease and has been used over more than two decades to gain insight into the physiopathology of DD and to develop new therapeutics (Tanaka et al. 2000; Manso et al. 2020). Recently, a new hypomorphic DD mouse model bearing an in-frame deletion of exon 6 was generated (129; 129S4/SvJae;129S6/SvEvTac; C57BL/6;SJL-*Lamp2*^tm1.2Ces^; MGI:7339154) (Alcalai et al. 2021). This mouse model only displayed a mild, predominant cardiac phenotype (Alcalai et al. 2021; Yadin et al. 2022, 2023). In contrast to the existing mouse models, LAMP2-deficient rats, created by TALEN Genome Editing Technology (Lamp2^y/−^ [SD-*Lamp2*^em1^; RRID: RGD_13703119]), showed greater similarity to DD patients in terms of onset and multisystemic lesions and highly mimicked the clinical differences observed by sex in human patients (Wang et al. 2017).

One of the main hallmarks of DD is heart dysfunction that progresses to heart failure as disease worsens (D’souza et al. 2014). LAMP2 deficient mice showed a depressed cardiac contractile function resulting in an attenuated cardiac pump reserve likely to be caused by autophagic vacuoles in LAMP2 deficient myocytes (Stypmann et al. 2006). Furthermore, LAMP2-deficient mice exhibited cardiomegaly, which may be secondary to the insufficient contractile function of the heart muscle (Tanaka et al. 2000; Saftig et al. 2001; Stypmann et al. 2006). Although the KO mouse model developed severely reduced cardiac contractility, the cardiac phenotype in humans is more severe, and the cardiomyocyte vacuolation is more pronounced compared with LAMP2-deficient mice (Saftig et al. 2001; Endo et al. 2015). In this regard, *Lamp2*^*y/−*^ rats displayed damage to the myocardium with increased serum troponin I, myoglobin and creatinine kinase (CK), and not only exhibited thickened structure but abnormal heart function (Ma et al. 2018). Moreover, *Lamp2*^*y/−*^ rats presented more pronounced cardiomegaly than LAMP2-deficient mice (Ma et al. 2018). At microscopic level, *Lamp2*^*y/−*^ male rats show substantial myocyte disruption, hypertrophic muscle fibers, interstitial fibrosis and microvascular hyperplasia in the heart tissue (Ma et al. 2018).

Skeletal myopathy is another distinguishing characteristic of affected male DD patients (Zhai et al. 2023). *Lamp2*^*y/−*^ rats displayed skeletal muscle fiber disarray and interstitial fibrosis, with glycogen deposition and increased CK in serum (Ma et al. 2018). In contrast, LAMP2 deficient mice showed normal serum CK activity although the skeletal muscle presented fiber degeneration, fiber splitting and ring fibers (Tanaka et al. 2000). Moreover, *Lamp2*^*y/−*^ rats showed reduced exploration and impaired locomotion, compared to the unaltered gross motor behavior observed in LAMP2-deficient KO mice (Tanaka et al. 2000; Rothaug et al. 2015; Ma et al. 2018). Therefore, the pathological changes observed in skeletal muscle along with impaired locomotor activity of *Lamp2*^*y/−*^ rats demonstrated that the skeletal muscle phenotype of affected rats was more exacerbated compared to KO mice.

An AAV-gene therapy approach based on a single intravenous administration of AAV9 vectors coding for the human LAMP2B isoform (AAV9-LAMP2B) was developed in the KO mouse model, since the DD rat model was not generated yet (Manso et al. 2020). Dose-dependent restoration of human LAMP2B protein in the heart, liver, and skeletal muscle tissue, together with an improvement of the autophagic flux was obtained in AAV9-LAMP2B-treated KO mice (Manso et al. 2020). Moreover, cardiac function was improved, and transaminases were reduced, indicating therapeutic benefit on heart and liver (Manso et al. 2020). Recently, these promising results allowed the clinical translation of this AAV gene therapy to DD patients (clinical trials NCT03882437 and NCT06092034).

### Maroteaux-Lamy syndrome

Maroteaux-Lamy syndrome, also known as Mucopolysaccharidosis VI (MPSVI), is a rare LSD with an autosomal recessive heritance caused by low to absent activity of the lysosomal enzyme arylsulfatase B (ASB). ASB catalyzes the degradation of the glycosaminoglycans (GAGs) dermatan sulfate (DS) and chondroitin sulfate (CS) in various tissues and organs of the body (Valayannopoulos et al. 2010). Clinically, MPSVI shows a wide spectrum of signs and symptoms among individuals ranging from rapidly to slowly progressing forms (Tomanin et al. 2018). The rapidly progressing form presents an onset from birth with severe symptoms, and death before the 2nd or 3rd decades (D’avanzo et al. 2021). In contrast, the slowly progressing forms have a later onset with milder symptoms and death in the 4th or 5th decades (D’avanzo et al. 2021). The classical clinical features of MPSVI patients are impairment of the osteoarticular system including growth retardation/short stature, coarse facial features, joint stiffness, degenerative joint disease, dysostosis multiplex and cervical cord compression (Tomanin et al. 2018; Nicolas-Jilwan 2020; D’avanzo et al. 2021).

Four MPSVI mouse models have been described (D’avanzo et al. 2021; Hosoba 2022; Hwang-Wong et al. 2023). The first KO mouse model was generated by targeted disruption of the *ArsB* gene (129P2/OlaHsd; C57BL/6J- *Arsb*^tm1Cptr^; MGI:2655540) (Evers et al. 1996). The lack of ARSB activity resulted in GAG accumulation in most tissues and organs (Evers et al. 1996; Strauch et al. 2003; Ferla et al. 2014, 2017; Alliegro et al. 2016). MPSVI KO mice presented overt facial dysmorphia, shortened and thickened long bones, and pelvic and costal abnormalities, and widened growth plate, loss of columnar arrangement of chondrocytes, and ballooned and vacuolated chondrocytes in the growth plate (Evers et al. 1996). Moreover, KO mice exhibited increased GAG content in heart valves and myocardium, decreased myocardial contraction, 50% reduction of cardiac output, and insufficiencies in the mitral and aortic valves (Strauch et al. 2003; Ferla et al. 2014, 2017; Alliegro et al. 2016). Overall, KO mice recapitulated the skeletal and cardiac pathological phenotypes of human MPSVI (Strauch et al. 2003).

A second MPSVI mouse model was obtained by mutagenesis using N-ethyl-N-nitrosourea (C57BL/6J-*Arsb*^m1J^/GrsrJ; MGI:3849442; RRID: IMSR_JAX:005598) (Curtain and Donahue 2009; D’avanzo et al. 2021). *Arsb*^*m1J/m1J*^ mice were smaller, showing decreased adiposity and higher lean mass than WT (Curtain and Donahue 2009). *Arsb*^*m1J/m1J*^ mice also exhibited shortened skull length with reduced bone mineral density and bone mineral content, shortened snout and upper jaw length, wide-set eyes, thicker tail and shortened limbs (Curtain and Donahue 2009; Pohl et al. 2018). Tracheal cartilage and femoral growth plate was disorganized and thickened (Curtain and Donahue 2009; Entchev et al. 2020).*Arsb*^*m1J/m1J*^ mice recapitulated key pathological features of MPSVI.

KI MPSVI mice bearing the human missense mutation p. Y85H (c. 252T > C), which has been reported in Japanese patients with severe phenotypes, were generated by CRISPR-Cas9 technology (C57BL/6J-*Arsb*^em1^) (Tomanin et al. 2018; Hosoba 2022). MPSVI KI mice exhibited a phenotype similar to that of patients with the same mutation. The absence of enzyme activity resulted in reduced body weight as soon as 4 months of age, characteristic facial features and shortened bone size at 10 months of age (Hosoba 2022).

Another MPSVI mouse model was generated using the conditional by inversion-based (COIN-based) method (*Arsb*^*COIN/COIN*^ [C57BL/6 N;129S6/SvEv-*Arsb*^tm1Kdc^]) (Hwang-Wong et al. 2023). In this model, a region of murine *Arsb* encompassing exon 5 along with flanking intronic sequences was placed in the antisense orientation (inverted), rendering *Arsb* null (Hwang-Wong et al. 2023). *Arsb*^*COIN/COIN*^ mice presented elevated GAG content in liver, heart, and kidney; and reduced body weight, cranial length/width ratios and skeletal size with long bones remarkably shorter.

Although these mouse models showed a similar osteoarticular phenotype to that of MPSVI patients, the musculoskeletal pathology was better recapitulated in MPSVI rats. The naturally occurring MPSVI rat model (IS-*Arsb*^m1J^) was first detected in 1988 in the Ishibashi hairless rat strain and a colony was then established to characterize the clinical, pathological, and biochemical features (Yoshida et al. 1993b). The 507insC mutation, which resulted in a frame shift and premature termination at codon 258, was the causative mutation in the MPSVI rat model (Kunieda et al. 1995). The ARSB deficiency (< 5% of enzymatic activity) increased urinary excretion of GAGs, in particular, DS and GAG accumulation in several organs and tissues (Yoshida et al. 1993a, b; Simonaro et al. 1997; Tessitore et al. 2008, 2009; Saccone et al. 2014; Frohbergh et al. 2014).

As soon as 3 weeks of age, MPVI rats presented an evident facial dysmorphia with a wide, short cranium, thick, coarse zygomatic arches, and thickened bullae and temporal bones (Yoshida et al. 1993b; Simonaro et al. 1997). In addition, MPSVI rats displayed severe dysostosis multiplex, exhibiting smaller bodies, shorter limbs (long bones) and shorter and thicker tails (Yoshida et al. 1993b). Moreover, affected rats presented shorter and narrower cervical vertebrae, short and coarse clavicles, short, wide ribs and thickened and abnormal, collapsed trachea (Yoshida et al. 1993b; Simonaro et al. 1997; Eliyahu et al. 2011; Schuchman et al. 2013). Structural, compositional, and functional biomechanical alterations found in lumbar spines of MPSVI rats are known to be associated with mechanical back pain, commonly found in MPSVI patients (Lai et al. 2013). Abnormalities in MPSVI rats included enhanced apoptosis of articular chondrocytes, excessive proliferation of MPS synovial fibroblasts, progressive loss of histological architecture and disorganization of growth plates contributing to the abnormal bone formation (Simonaro et al. 2005, 2008; Frohbergh et al. 2014; Guevara-Morales et al. 2020). The major impairment of the osteoarticular system resulted in gait impairment, reduced hanging strength, impaired vertical exploratory ability, and reduced locomotor activity and endurance (Eliyahu et al. 2011; Saccone et al. 2014; Frohbergh et al. 2014).

The MPSVI rat model played a crucial role in the early development of an AAV-based gene therapy for MPSVI. Newborn MPSVI rats treated intravenously with AAV8 vectors expressing the ARSB gene showed lessening of skeletal abnormalities, reduced urinary and tissue GAGs storage, and corrected inflammation and apoptosis (Tessitore et al. 2008). Subsequent studies in other animal models, particularly MPSVI cats and MPSVI KO mice, provided valuable insights into efficacy, safety, and optimal treatment strategies (Cotugno et al. 2011; Ferla et al. 2013, 2014, 2021; Alliegro et al. 2016). These pre-clinical findings paved the way for successful clinical translation. Specifically, the Phase I/II clinical trial in MPSVI patients (NCT03173521) demonstrated the safety and efficacy of this gene therapy, with sustained ARSB protein levels for at least 3 years (Brunetti-Pierri et al. 2022; Rossi et al. 2024).

### Morquio A syndrome

Morquio A syndrome, also known as Mucopolysaccharidosis type IVA (MPSIVA), is a rare LSD with autosomal recessive inheritance caused by mutations in N-acetyel-galactosamine-6-sulfate sulfatase (*GALNS*) gene (Neufeld and Muenzer 2001). In MPSIVA patients, the deficiency of GALNS results in progressive pathological accumulation of GAGs keratan sulfate (KS) and chondroitine-6-sulfate (C6S) in several tissues, mainly in the skeletal system, the cartilaginous tissues, cornea and heart valves (Neufeld and Muenzer 2001; Sawamoto et al. 2016; Khan et al. 2017; Melbouci et al. 2018). Morquio A patients appear normal at birth, but over time they show short stature and skeletal dysplasia, as bone deformity is one of the most common initial symptoms of the disease (Montaño et al. 2007; Byers et al. 2018; Sawamoto et al. 2020). The undegraded KS and C6S, mainly localized in chondrocytes and extracellular matrix of cartilage, induces abnormal chondrogenesis and endochondral ossification, leading to poor bone mineralization and abnormal growth (Montaño et al. 2007; Sawamoto et al. 2016; Byers et al. 2018). Most affected patients need multiple surgical procedures including spinal decompression/fusion, leg osteotomies, hip reconstruction/replacement, and/or corrective tracheal surgery (Sawamoto et al. 2020). Non-skeletal manifestations in MPSIVA patients mainly affect the respiratory, the cardiovascular and the digestive system, as well as alterations in visual and auditory function (Northover et al. 1996; Montaño et al. 2007; Tomatsu et al. 2011; Khan et al. 2017; Sawamoto et al. 2020). Respiratory failure and cardiovascular disease in combination with cervical spinal cord complications are the main cause of death in affected patients around the 2nd or 3rd decade of life (Lavery and Hendriksz 2015).

To date, three different mouse models of Morquio A syndrome have been generated. The KO mouse model was generated by deletion of part of intron 1 and the whole exon 2 of the *Galns* gene resulting in a frameshift mutation (*Galns*^*-/-*^ mice [129 × 1SvJ; C57BL/6J-*Galns*^tm1Toma^; MGI:2686939]) (Tomatsu et al. 2003). A KI mouse was obtained by inserting the p.C76S point mutation, a highly conserved amino acid key in the active center of the protein, in the endogenous murine *Galns* gene by targeted mutagenesis (129 × 1SvJ; C57BL/6J-*Galns*^tm3Toma^; MGI:3715448) (Tomatsu et al. 2007). In the third model, the missense mutation p.C76S was introduced in exon 2 and, additionally, a copy of an inactive human GALNS coding sequence (p.C79S) was introduced by direct mutagenesis in the adjacent intron 1 to generate a humanized MPSIVA mouse model (129 × 1SvJ; C57BL/6J-*Galns*^tm2(GALNS)Toma^; MGI:3616632) (Tomatsu et al. 2005a).

Although all these mouse models showed undetectable levels of GALNS activity and increased lysosomal GAG accumulation and distension in multiple organs and tissues, none of them exhibited the characteristic bone pathology of human patients (Tomatsu et al. 2003, 2005a, 2007). This lack of bone phenotype is thought to be due to differences in KS synthesis in bone between humans and mice (Venn and Mason 1985; Tomatsu et al. 2007) and to the fact that chondroitin 4-sulfate is mainly present in the growth plate cartilage of mice in contrast to human growth cartilage, in which chondroitin 6-sulfate is highly present (Gaffen et al. 1995; Rowan et al. 2013). To overcome the limitations of MPSIVA mouse models, a rat model of Morquio A was generated by CRISPR/Cas9 technology (SD-*Galns*^em1Fbos^) (Bertolin et al. 2021). MPSIVA rats bear the most frequent and severe human missense mutation c.1156 C > T (p.R386C) (Tomatsu et al. 2005b), leading to undetectable levels of GALNS activity in all tissues analyzed (Bertolin et al. 2021). At birth, no gross morphological alterations were observed in MPSIVA rats which weighed similar to WT littermates. Starting at 1 month of age, MPSIVA rats showed decreased body weight gain and pathological accumulation of KS in liver and serum (Bertolin et al. 2021). Over time, they presented with a reduction in naso-anal and tibial length, indicative of reduced body size and the involvement of skeletal pathology (Bertolin et al. 2021). Increased KS content was detected in some key peripheral tissues and organs, such as liver, adipose tissue and bones, as well as serum (Bertolin et al. 2021). In agreement with reduced body size, affected rats showed reduced growth plate area and calcification zone, in conjunction with alterations in trabecular and compact bone composition, evidencing alterations in ossification as rats aged (Bertolin et al. 2021). MPSIVA rats presented with hypertrophic chondrocytes loaded with vacuoles of undegraded substrates in bones (Bertolin et al. 2021). Consistent with joint alterations, MPSIVA rats showed reduced grip strength (Bertolin et al. 2021). Similar to humans, MPSIVA rats showed dental malocclusion, fragility, and enamel hypoplasia (Bertolin et al. 2021). In the lungs of MPSIVA rats, tracheal hyaline cartilage, respiratory epithelium, and lamina propria were altered with the presence of storage pathology in chondrocytes, ciliary cells, and fibroblasts, respectively (Bertolin et al. 2021). In addition, MPSIVA rats exhibited the main cardiac abnormalities of Morquio A patients, including mitral valve dysfunction and aorta wall alterations, that contributed to increased heart rate (Tomatsu et al. 2011; Yasuda et al. 2013; Kampmann et al. 2016; Bertolin et al. 2021). Overall, MPSIVA rats recapitulated the main hallmarks of Morquio A syndrome, including skeletal dysplasia caused by alterations in endochondral ossification resulting from abnormal chondrogenesis (Tomatsu et al. 2011, 2014; Bertolin et al. 2021). Furthermore, MPSIVA rats exhibited the most severe non-skeletal manifestations observed in MPSIVA patients, such as dental, respiratory and cardiovascular complications (Lavery and Hendriksz 2015; Peracha et al. 2018; Bertolin et al. 2021).

Treatment of MPSIVA rats with AAV9 vectors encoding *Galns* (AAV9-*Galns*) resulted in widespread transduction of bones, cartilage and all peripheral tissues mainly affected in Morquio A disease (Bertolin et al. 2021). This led to long-term (1 year) increase of GALNS activity in liver and serum and whole-body correction of KS levels, thus preventing body size reduction, skeletal dysplasia and cartilage deterioration (Bertolin et al. 2021). Noticeably, counteraction of tracheal, dental and cardiac pathology was also observed (Bertolin et al. 2021). These findings highlighted the therapeutic potential of AAV9-*Galns* gene therapy to correct the severe whole-body alterations found in MPSIVA patients and supported its future clinical translation. Furthermore, the long-term durability of the approach in conjunction with the excellent therapeutic efficacy laid the foundation for the treatment of other diseases with skeletal involvement.

## Conclusions

Overall, the current core of evidence reveals that rat models of LSDs, particularly Pompe and Danon diseases, and Maroteaux-Lamy and Morquio A syndromes, more accurately recapitulate the severe phenotypes observed in human patients compared to existing mouse models. The PD rat model exhibits earlier onset and more rapid progression of muscle pathology, including glycogen accumulation, muscle weakness, and impaired function, as well as earlier cardiomegaly and reduced lifespan. This model closely mimics the human IOPD phenotype. Similarly, rat models of Danon disease, and Maroteaux-Lamy and Morquio A syndromes closely reproduce the cardiac, skeletal muscle, and bone and joint alterations characteristic of these diseases in human patients. These findings suggest that rat models with musculoskeletal involvement provide more clinically relevant insights for studying LSD pathophysiology. Moreover, rat models are key for the assessment of efficient AAV-mediated gene therapies for these diseases.

## Data Availability

No datasets were generated or analysed during the current study.
